# Clinicians’ knowledge and understanding regarding multidisciplinary treatment implementation: a study in municipal public class III grade A hospitals in Southwest China

**DOI:** 10.1186/s12909-023-04891-0

**Published:** 2023-12-04

**Authors:** Xuemin Zhong, Xianbao Zeng, Longchao Zhao, TaoChen, Xing Min, Rui He

**Affiliations:** 1 Chengdu Second People’s Hospital, Postal Address: No.10 Qingyunnan Street, Jinjiang District, Chengdu, Sichuan China; 2 Women and Children’s Hospital of Chongqing Medical University, Chongqing, China; 3 Chongqing Health Center for Women and Children, Chongqing, China

**Keywords:** Understanding, Multidisciplinary treatment, Multidisciplinary consultations, Clinicians

## Abstract

**Background:**

Previous studies have highlighted several problems in the implementation of multidisciplinary treatment (MDT) from a managerial perspective. However, no study has addressed these issues from clinicians’ perspective. Therefore, this study aimed to identify and address the existing problems in MDT by investigating what clinicians know and think about the implementation of MDT.

**Methods:**

A self-designed questionnaire was used to evaluate clinicians’ understanding of MDT in municipal public Class III Grade A hospitals in Western China using a cross-sectional questionnaire study.

**Results:**

Overall, 70.56% of clinicians knew the scope of MDT, and 63.41% knew the process of MDT. Professional title (*P* = 0.001; OR: 2.984; 95% CI: 1.590–5.603), participated in MDT (*P* = 0.017; OR: 1.748; 95% CI: 1.103–2.770), and application for MDT (*P* = 0.000; OR: 2.442; 95% CI: 1.557–3.830) had an impact on clinicians’ understanding of the scope of MDT. Professional title (*P* = 0.002; OR:2.446; 95% CI: 1.399–4.277) and participation in MDT (*P* = 0.000; OR: 2.414; 95% CI: 1.581–3.684) influenced clinicians’ understanding of the scope of MDT. More than 70% of the respondents thought that MDT was important in medical care. However, less than half of the clinicians who had attended MDT were currently satisfied with the results of MDT.

**Conclusion:**

Most clinicians agreed that MDT was crucial in clinical care. However, more than a third of clinicians did not fully understand the scope and process of MDT. Appropriate measures are necessary to improve the quality of MDT. Our study suggests that healthcare administration should strengthen MDT education, especially for new and young clinicians.

**Supplementary Information:**

The online version contains supplementary material available at 10.1186/s12909-023-04891-0.

## Background

With the advancement of modern medical technology and the deepened understanding of diseases, diagnosing and treating complex diseases independently within a single specialty is increasingly challenging [1]. This necessitates the collaboration of multiple disciplines within the hospital setting [2]. Multidisciplinary treatment (MDT) involves gathering experts from various fields to conduct whole-process diagnosis, treatment planning, and continuous treatment for patients with rare or complex diseases [3]. MDT originated in the United States and has been widely adopted in countries such as the United Kingdom, France, Germany, Japan, and Australia. It has gradually gained acceptance worldwide and has demonstrated positive therapeutic outcomes [4–10]. In China, the application of MDT has expanded beyond tumor treatment to now encompass challenging diseases across various departments in general hospitals [11–13].

MDT can be categorized into two primary forms. The first form involves a clinical diagnosis and treatment model where experts from two or more related disciplines form a relatively fixed expert group. They regularly convene to discuss and propose diagnoses and treatment opinions for diseases affecting specific organs or systems. This model is commonly implemented in high-level oncology hospitals or oncology departments within large general hospitals [11]. It represents a leading international standardized diagnosis and treatment model [14–17]. The second form refers to multidisciplinary consultations organized by relevant departments according to the needs of patients or their families. Large general hospitals commonly adopt this form of consultation for difficult and critical cases [18].

The importance of MDT has been acknowledged in the "Evaluation Standards for Tertiary Hospitals (2022 edition)" issued by the National Health Commission of China, where MDT is a core component of medical quality and safety [19]. Therefore, standardizing the MDT process and implementing a high-quality MDT have become top priorities in the construction of tertiary hospitals [20]. Previous studies have highlighted various issues in the implementation of MDT from the perspective of managers, such as the uneven level of participation among doctors, inadequate preparation before applying for departments, and a lack of collaborative spirit among participating departments [21]. Alhough clinicians are the protagonists participating in the MDT, there is a lack of studies addressing these existing problems from their perspective.

Therefore, this study aims to investigate clinicians’ understanding and thinking of MDT and the factors influencing their understanding of the same. The findings of this study will contribute to the field by providing valuable insights from clinicians’ perspectives, ultimately leading to better implementation of MDT.

## Methods

### The questionnaire design

Based on an extensive literature review, we collaborated with four experienced medical management experts and two epidemiologists who specialize in statistics to design a questionnaire investigating how clinicians’ understand and think of MDT. Informed consent was obtained from all the participants, and confidentiality was guaranteed.

We conducted a preliminary survey and incorporated feedback from respondents to modify and improve the questionnaire. The final version comprised 25 questions, including single-choice and multiple-choice options. The first section consisted of seven general questions regarding sex, age, education, department, and professional title (junior, intermediate, and senior titles). After a certain period of assessment, doctors with different educational degrees are promoted from junior to intermediate level, and finally promoted to senior level. The second section explored the participants’ understanding and views of MDT. Finally, experts evaluated the questionnaire to ensure its reliability and validity. The questionnaire’s reliability was assessed using the Cronbach’s-α-coefficient, which yielded a value of 0.921. The questionnaire is provided as Supplementary material 1.

### Data collection

Data collection was conducted using WeChat (2012 edition, Changsha Ranxing Technology Information Co., Ltd.). We selected seven public Grade A general hospitals in Sichuan and Chongqing through convenience sampling. Consent was obtained in advance from the medical department of each hospital, and the head of each medical department subsequently shared the questionnaire’s QR code with the clinical department director. The questionnaire was then distributed to each clinical department through their respective department directors, who invited the doctors in those departments to participate. The survey was conducted between June 8 and June 18, 2023.

### Statistical analysis

The participants’ responses were exported to Microsoft Excel for data classification and analysis. The reliability of the questionnaire was assessed using Cronbach’s-α test, while the validity was analyzed through principal component analysis using exploratory factor analysis. All statistical analyses were performed using R (version 4.3.1). Continuous variables were presented as medians and interquartile ranges, whereas categorical variables were presented as numbers and percentages. We used the Wilcoxon rank-sum test (for skewed distributions) or Chi-square test (for categorical variables) to test differences in characteristics between awareness of the scope and process of multidisciplinary consultation.

We utilized logistic regression to explore the factors influencing clinician’s understanding of MDT. The threshold for statistical significance was set at *P* < 0.05. The association between the title level, position, familiarity with the scope, and MDT process was visualized using a Sankey diagram.

## Results

### Basic information of respondents

In this online survey, 594 questionnaires were collected, of which 574 were found to be valid after removing 21 duplicate and invalid responses. The mean age of the respondents was 36 years (31–43), with men accounting for 41.29%. Among the respondents, there were 120 department deputies/directors, 50 medical team leaders, and 404 general doctors (Table 1).

**Table 1:** Basic information of respondents

Value	Scope of MDT				The progress of MDT			
	Fully aware (%)	Partially aware (%)	Not aware (%)	*P*	Fully aware (%)	Partially aware (%)	Not aware (%)	*P*
Sex								
Male	175 (73.84)	51 (21.52)	11 (4.64)	0.073	156 (65.82)	65 (27.43)	16 (6.75)	0.493
Female	230 (68.25)	98 (29.08)	9 (2.67)		208 (61.72)	108 (32.05)	21 (6.23)	
Age M (Q_25_,Q_75_)	37 (32,45)^ab^	33 (30,38)^a^	32 (28.25,35.75)^b^	< 0.001	37 (32,44)^ab^	34 (30,41)^a^	32 (29,36.5)^b^	< 0.001
Educational background								
College	7 (63.64)	3 (27.27)	1 (9.09)	0.60 6	5 (45.45)	5 (45.45)	1 (9.09)	0.078
Bachelor	146 (75.26)	41 (21.13)	7 (3.61)		122 (62.89)	54 (27.84)	18 (9.28)	
Master	213 (68.05)	90 (28.75)	10 (3.19)		194 (61.98)	104 (33.23)	15 (4.79)	
Doctor	39 (69.64)	15 (26.79)	2 (3.57)		43 (76.79)	10 (17.86)	3 (5.46)	
Professional title								
Junior	77 (58.78)^ab^	48 (36.64)^a^	6 (4.58)^b^	< 0.001	68 (51.91)^a^	53 (40.56)^a^	10 (7.63)	< 0.001
Intermediate	170 (67.46)^cd^	70 (27.78)^c^	12 (4.76)^d^		155 (61.51)	76 (30.16)	21 (8.33)	
Senior	158 (82.72)^abcd^	31 (16.23)^ac^	2 (1.05)^bd^		141 (73.82)^a^	44 (23.03)^a^	6 (3.14)	
Duty								
Yes	148 (87.10)^ab^	22 (12.90)^a^	0 (0.00)^b^	< 0.001	135 (79.4)^ab^	33 (19.4)^a^	2 (1.2)^b^	< 0.001
No	257 (63.61)^ab^	127 (31.44)^a^	20 (4.95)^b^		229 (56.68)^ab^	140 (34.65)^a^	35 (8.66)^b^	
Participation in MDT								
Yes	327 (76.9)^ab^	89 (20.9)^a^	9 (2.1)^b^	< 0.001	296 (69.6)^ab^	113 (26.6)^a^	16 (3.8)^b^	< 0.001
No	78 (52.3)^ab^	60 (40.3)^a^	11 (7.4)^b^		68 (45.6)^ab^	60 (40.3)^a^	21 (14.1)^b^	
Ever applied for MDT								
Yes	286 (78.8)^ab^	67 (18.5)^a^	10 (2.8)^b^	< 0.001	265 (73.0)^ab^	82 (22.6)^a^	16 (4.4)^b^	< 0.001
No	119 (56.4)^ab^	82 (38.9)^a^	10 (4.7)^b^		99 (46.9)^ab^	91 (43.1)^a^	21 (10.0)^b^	

*Note*: Senior titles include associate seniors

Participants with duty include the department director, deputy director, and medical team leader

Where the letter “^abcd^” is superscript, it indicates statistical differencebetween the two compared groups

### Clinician’s understanding of MDT scope and process

Among all respondents, 70.56% of clinicians were aware of the scope of MDT, whereas 20 were unaware. Regarding the MDT process, 364 clinicians were knowledgeable, whereas 37 had no awareness of the procedure.

### Factors influencing clinicians’ understanding of MDT

**Fig. 1:**
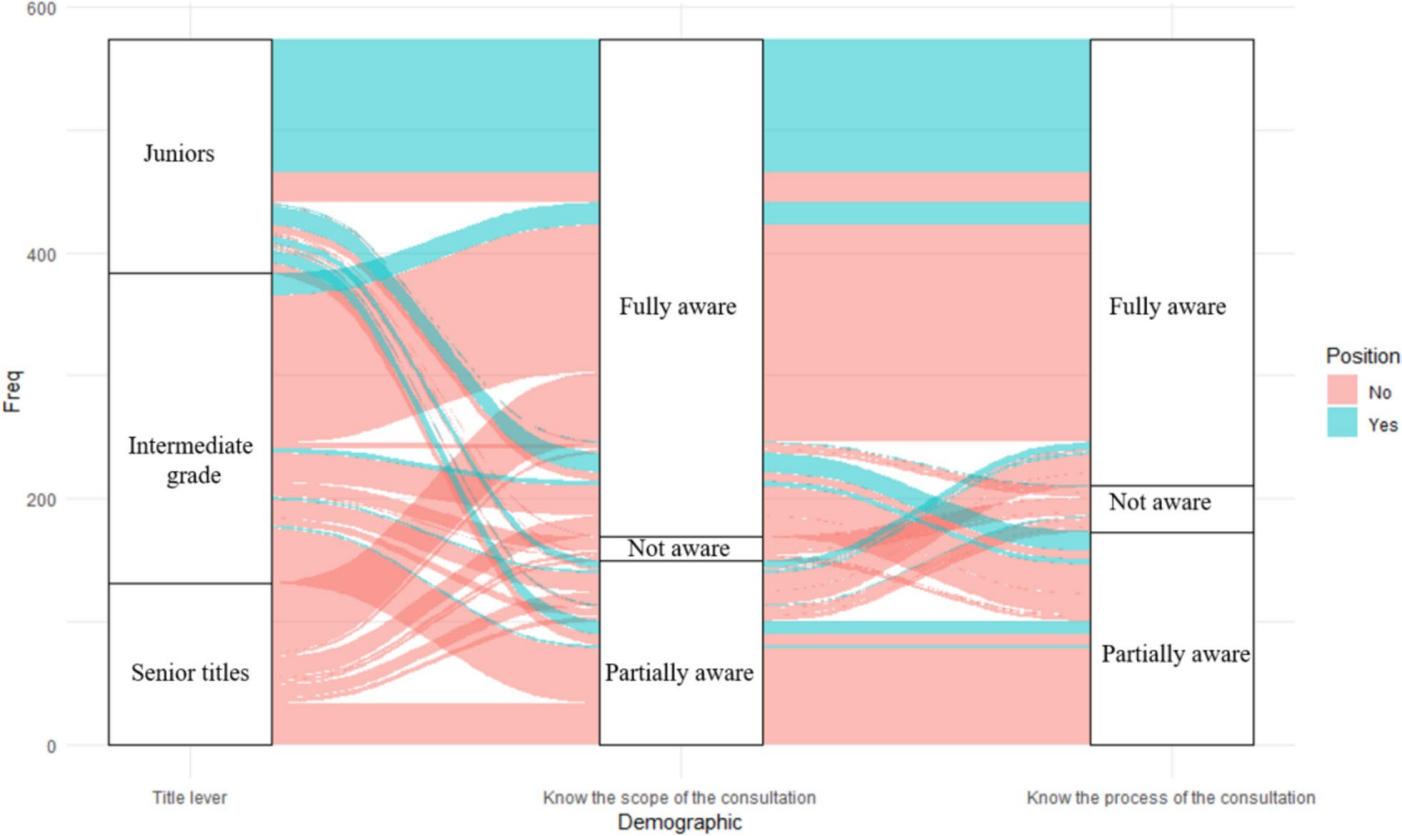
The association between the title level, position, knowledge of the scope, and the process of MDT

Age, professional title, duty, participation in MDT, and whether they had applied for MDT had a significant impact on clinicians’ understanding of the MDT scope and process (*P* < 0.05). Among these factors, professional title and duty showed a positive correlation with the understanding of the MDT process and scope (Fig. 1).

The result represented in Table 1 indicate no statistically significant difference between the partially aware group and the not aware group for each variable. Therefore, in the multivariate logistic regression, we combined clinicians who were partially aware and those not aware of the MDT scope and process for analysis. The results suggest that professional title (*P* = 0.001; OR: 2.984;95% CI: 1.590–5.603), participation in MDT (*P* = 0.017; OR: 1.748; 95% CI: 1.103–2.770), ever applied for MDT (*P* = 0.000; OR: 2.442; 95% CI: 1.557–3.830) have an impact on clinicians’ understanding of MDT scope (Table 2). Additionally, professional title (*P* = 0.002; OR: 2.446; 95% CI: 1.399–4.277) and ever applied for MDT (*P* = 0.000; OR: 2.414; 95% CI: 1.581–3.684) had an impact on clinicians’ understanding of the MDT scope (Table 3).

**Table 2:** Multivariate analysis of factors affecting clinicians’ awareness of MDT scope

Variable	B	SE	Wals	*P*	Odds ratio	95%Confidence interval	
						Lower	Upper
Sex	-0.023	0.205	0.013	0.909	0.977	0.654	1.460
Age	0.004	0.020	0.041	0.840	1.004	0.966	1.044
Educational background	-0.058	0.218	0.071	0.790	0.944	0.616	1.445
Professional title	1.093	0.321	11.575	0.001	2.984	1.590	5.603
Duty	-0.292	0.385	0.573	0.449	0.747	0.351	1.590
Participation in MDT	0.558	0.235	5.650	0.017	1.748	1.103	2.770
Ever applied for MDT	0.893	0.230	15.117	0.000	2.442	1.557	3.830

**Table 3:** Multivariate analysis of factors affecting clinicians’ awareness of MDT progress

Variable	B	SE	Wals	*P*	Odds ratio	95%Confidence interval	
						Lower	Upper
Sex	-0.122	0.192	0.403	0.525	0.885	0.607	1.29
Age	-0.006	0.017	0.112	0.738	0.994	0.961	1.029
Educational Background	0.056	0.202	0.076	0.783	1.057	0.712	1.569
Professional title	0.895	0.285	9.849	0.002	2.446	1.399	4.277
Duty	-0.147	0.351	0.176	0.675	0.863	0.433	1.719
Participation in MDT	0.413	0.229	3.251	0.071	1.512	0.965	2.37
Ever involved in MDT	0.881	0.216	16.683	0.000	2.414	1.581	3.684

### Clinician participation in MDT

In the survey, 425 physicians (74%) participated in MDT. The four main reasons for their participation were: unclear or difficult diagnosis and treatment in the department, diseases involving multi-organ or multi-system lesions requiring assistance from multi-department, clear diagnosis with long-term treatment effect, and the tendency of medical disputes or the need to consult key patients. The average duration of an MDT session is typically 30–60 min. Among the consultants, 50.35% often followed-up on the patients’ prognosis after MDT. Among the doctors who participated in the survey, 63.2% had applied for MDT. The main reason for applying was similar to those mentioned earlier: unclear or difficult diagnosis and treatment, diseases involving multi-organ or multi-system lesions requiring assistance from multi-department, and clear diagnosis with poor long-term treatment effect. Medical disputes or the need to consult key patients were also important factors (Table 4).

**Table 4:** Clinician participation in MDT

Value	N(%)
Whether participated in MDT	
Yes	425 (74)
No	149 (26)
Main reasons for participating in MDT (multiple choices) (*N* = 425)	
Unclear diagnosis or difficulty in diagnosis and treatment in the department	353 (83.06)
The diagnosis was clear, but the long-term treatment effect was poor	184 (43.29)
The disease involved multi-organ and multi-system lesions requiring multi-department assistance	367 (86.35)
The perioperative period is associated with multiple diseases and requires risk assessment	108 (25.41)
Suspected acute infectious disease	8 (1.88)
Emergency patients with difficult and critical cases cannot be diagnosed in time, affecting rescue	99 (23.29)
Patients who are prone to medical disputes or certain key patients	156 (36.71)
The general duration of MDT (*N* = 425) (min)	
< 30	62 (14.58)
30–60	307 (72.24)
60–120	50 (11.76)
> 120	6 (1.41)
Whether often followed up the prognosis of the patients after MDT	
Always	214 (50.35)
Sometimes	188 (44.24)
Never	23 (5.41)
Whether applied for MDT	
Yes	363 (63.2)
No	211 (36.8)
Main reasons for applying for MDT (multiple choices) (*N* = 363)	
Unclear diagnosis or difficulty in diagnosis and treatment in the department	299 (82.37)
Clear diagnosis with poor but long-term treatment effect	158 (43.53)
The disease involved multi-organ and multi-system lesions requiring multi-department assistance	301 (82.92)
The perioperative period is associated with multiple diseases and requires risk assessment	82 (22.59)
Suspected acute infectious disease	9 (2.48)
Emergency patients with difficult and critical cases cannot be diagnosed in time, affecting rescue	87 (23.97)
Patients who are prone to medical disputes or certain key patients	153 (42.15)

### Clinician’s view on MDT

More than 70% of the respondents believed that MDT plays a considerably important role in improving medical quality and efficiency, reducing repeated consultations, improving patient satisfaction, and optimizing doctors’ diagnoses and treatment. However, only 48.94% of the clinicians who participated in the consultation were satisfied with MDT, and merely 49.58% of the physicians who applied for MDT were satisfied with the results. Among the respondents, only 48.3% believed that the hospital’s MDT was well implemented. The reasons and suggestions for clinicians’ dissatisfaction with MDT are shown in Table 5.

**Table 5:** Clinician’s view on MDT

Clinician’s view	N(%)
Effect of MDT on improving medical quality	
Important	416 (72.5)
Relatively important	143 (24.9)
Unimportant	15 (2.6)
Effect of MDT on reducing repeated consultation	
Important	432 (75.3)
Relatively important	131 (22.8)
Unimportant	11 (1.9)
Effect of MDT on controlling medical expenses	
Important	304 (53.0)
Relatively important	230 (40.10)
Unimportant	40 (7.0)
Effect of MDT on improving medical efficiency	
Important	429 (74.7)
Relatively important	135 (23.5)
Unimportant	10 (1.7)
Effect of MDT on improving patients’ satisfaction	
Important	473 (82.4)
Relatively important	97 (16.9)
Unimportant	4 (0.7)
Effect of MDT on improving doctors' diagnosis and treatment	
Important	555 (96.7)
Relatively important	19 (3.3)
Unimportant	0 (0.0)
Whether satisfied with MDT (*N* = 425)	
Always	208 (48.94)
Sometimes	214 (50.35)
Rarely	3 (0.71)
Reasons for dissatisfaction with MDT (multiple choices) (*N* = 217)	
The request for consultation was rushed and the consultant did not have enough time to prepare	91 (41.94)
Inadequate case preparation for consultation	128 (58.99)
The doctor who presided over the consultation was not able to synthesize the expert opinions and put forward feasible administrative and treatment plans	74 (34.10)
Many consultations have no practical meaning, and are merely a form of explanation to the patient or their family	122 (56.22)
Whether satisfied with the results of MDT (*N* = 363)	
Always	180 (49.58)
Sometimes	181 (49.86)
Rarely	2 (0.55)
Reasons for dissatisfaction with the results of MDT (multiple choices) (*N* = 183)	
Clinicians cannot attend the consultation on time	116 (63.39)
Consultant is not qualified enough to provide guidance for the patient	108 (59.02)
The participating departments lacked collaborative spirit	127 (69.40)
For some patients who have disputes or hidden dangers of arrears. The Consultation department is not willing to take the risk	98 (53.55)
Implementation of MDT	
Going well	277 (48.3)
General	270 (47.0)
Poor	27 (4.70)
Suggestions on MDT	
Control the request for consultation and give the consultation department sufficient time to prepare	410 (71.4)
Improve the professional ability of consultation and strict the qualifications of consultation	402 (70.0)
Strengthen preparation before consultation	360 (62.7)
Formulate measures to ensure timely consultation rate	297 (51.7)
Develop a consultation tracking system, supervise the implementation of diagnosis and treatment measures after consultation, track patient outcomes	393 (68.5)

## Discussion

Our study revealed that approximately one-third of clinicians lacked full knowledge of the consultation process. Doctors with greater clinical work experience or who participated in MDT demonstrated better understanding of the consultation process. However, the dissemination of MDT knowledge is inadequate. Therefore, medical management departments should strengthen their MDT knowledge, particularly among new and young doctors.

Only half of the doctors who participated in the consultation regularly followed up on the patient’s diagnoses and treatments. Many diseases exhibit typical manifestations only during certain stages of development, and some typical characteristics are fleeting. Therefore, a comprehensive system of dynamic thinking methods is necessary for observing and analyzing a disease. Consequently, the consultation result must be followed up and observed, incorporating lessons from previous cases [4]. Previous studies have recommended implementing a system for tracking consultation outcomes and engaging in retrospective case discussions to improve the diagnostic and differential abilities of specialists [4, 22–26].

In addition to difficult and complicated diseases, a significant proportion of MDT cases arise owing to disputes. To mitigate the increasingly intensified contradictions between clinicians and patients, medical personnel resort to MDT to minimize risks. However, this results in a large number of unnecessary consultations that burden clinicians and compromise the quality of medical care [4]. The study emphasizes the need for strict control over the application of MDT by medical management departments to prevent the wastage of medical resources. It strictly prohibits departments from using MDT to evade patients, as this wastes medical resources and diminishes the enthusiasm of participating departments.

Consistent with previous studies, our study found that most clinicians agreed that MDT can improve the quality and efficiency of care, reduce repeated consultations, and improve patient satisfaction [4, 27, 28]. However, only half of the clinicians expressed satisfaction with MDT. Some studies suggest that hospitals should establish an internet-based consultation platform, incorporate MDT modules into electronic medical records, and standardize MDT applications, case submissions, meeting minutes, and treatment follow-up [29].

Our study also had some limitations. The scope of the study is limited to a preliminary investigation in southwest China, which limits our results in terms of generalization.Second,as this survey is an electronic questionnaire,we did not get a response rate for the questionnaire.Third,the questionnaire survey we conducted was not as in-depth as an interview.Fourth,we classified aware as a group, partially aware and unaware as a group, with one group more than twice the size of the other in the analysis.This analysis may introduce sparsity and approximation errors. Fifth, the respondents’ answers about whether they are aware of MDT involve subjective self-judgment, and there is no possibility for an objective unified standard.

## Conclusion

Our study underscores the importance of MDT in clinical care. It highlights the need to enhance medical personnel’s understanding of MDT scope and process. We recommend further investigation into effective strategies for disseminating MDT knowledge among medical personnel, exploring the impact of enhanced MDT implementation on patient outcomes, and assessing the long-term benefits of comprehensive MDT systems across diverse healthcare settings. By addressing these aspects, the utilization of MDT can be optimized; this can ultimately improve the quality of patient care.

### Supplementary Information

**Additional file 1.**

## Data Availability

The datasets supporting the conclusions of this article are included as supplementary materials.

## Abbreviations

MDT: Mutidisciplinary treatment
CI: Confidence interval
OR: Odd ratio
